# Mechanisms of gut homeostasis regulating Th17/Treg cell balance in PMOP

**DOI:** 10.3389/fimmu.2024.1497311

**Published:** 2024-12-13

**Authors:** Peng Qi, Ruixuan Xie, Hao Liu, Zixuan Zhang, Yuan Cheng, Jilong Ma, Kangwei Wan, XingWen Xie

**Affiliations:** ^1^ Gansu University of Traditional Chinese Medicine, Lanzhou, China; ^2^ Wenzhou Medical University, Wenzhou, China; ^3^ Affiliated Hospital of Gansu University of Traditional Chinese Medicine, Lanzhou, China

**Keywords:** gut homeostasis, Th17/Treg cell balance, PMOP, mechanism study, gut microbiome

## Abstract

Postmenopausal osteoporosis (PMOP) is a metabolic bone disease driven by estrogen deficiency, primarily manifesting as reduced bone mass and heightened fracture risk. Its development is intricately linked to the balance between Th17 and Treg cells. Recent studies have highlighted the significant role of gut homeostasis in PMOP. The gut microbiota profoundly impacts bone health by modulating the host’s immune system, metabolic pathways, and endocrine functions. In particular, the regulation of Th17 and Treg cell balance by gut homeostasis plays a pivotal role in the onset and progression of PMOP. Th17 cells secrete pro-inflammatory cytokines that stimulate osteoclast activity, accelerating bone resorption, while Treg cells counteract this process through anti-inflammatory mechanisms, preserving bone mass. The gut microbiota and its metabolites can influence Th17/Treg equilibrium, thereby modulating bone metabolism. Furthermore, the integrity of the gut barrier is critical for systemic immune stability, and its disruption can lead to immune dysregulation and metabolic imbalances. Thus, targeting gut homeostasis to restore Th17/Treg balance offers a novel therapeutic avenue for the prevention and treatment of PMOP.

## 1 Introduction

PMOP is a metabolic bone disease primarily characterized by bone loss driven by estrogen deficiency. It progresses silently in postmenopausal women and is typically diagnosed only after the occurrence of fragility fractures (1). In China, approximately 20.6% of women over 40 years old are affected by osteoporosis (2). Gut homeostasis, a crucial regulator of bone metabolism, is strongly associated with the onset and progression of PMOP and has emerged as a key area of research (3). Gut homeostasis involves the stability of the gut microbiota, its metabolic functions, and the integrity of the gut’s physical and immune barriers (4). The gut microbiota, a vast and complex microbial community residing in the gastrointestinal tract, plays a pivotal role in bone metabolism and health by interacting with the host’s immune, metabolic, and endocrine systems (5).

The gut barrier consists of multiple layers (6): the outer layer, comprising mucus, symbiotic microbes, antimicrobial proteins, and secretory immunoglobulin A; the middle layer, formed by intestinal epithelial cells; and the innermost layer, which includes innate and adaptive immune cells (7). The gut mucosal immune system executes diverse immune defense functions through immune cells located at induction and effector sites (8). Within the gut, immune cells such as dendritic cells, macrophages, and T cells recognize and respond to microbiota and their metabolites, thereby regulating both local and systemic immune responses (9). Disruption of the gut barrier can result in epithelial cell apoptosis, promoting a pro-inflammatory environment that drives the differentiation of helper T cells 17 (Th17) and regulatory T cells (Treg) (10). Alterations in the gut microbiota are closely associated with changes in bone mass, particularly through their role in bone immunology and the gut-bone axis (11, 12). Previous studies have investigated the role of the gut microbiota in PMOP (13). Thus, elucidating the mechanisms by which gut homeostasis regulates the Th17/Treg balance could provide valuable insights into the pathogenesis of PMOP and inform the development of novel therapeutic strategies.

## 2 Pathological pathways of PMOP

**Figure 1:**
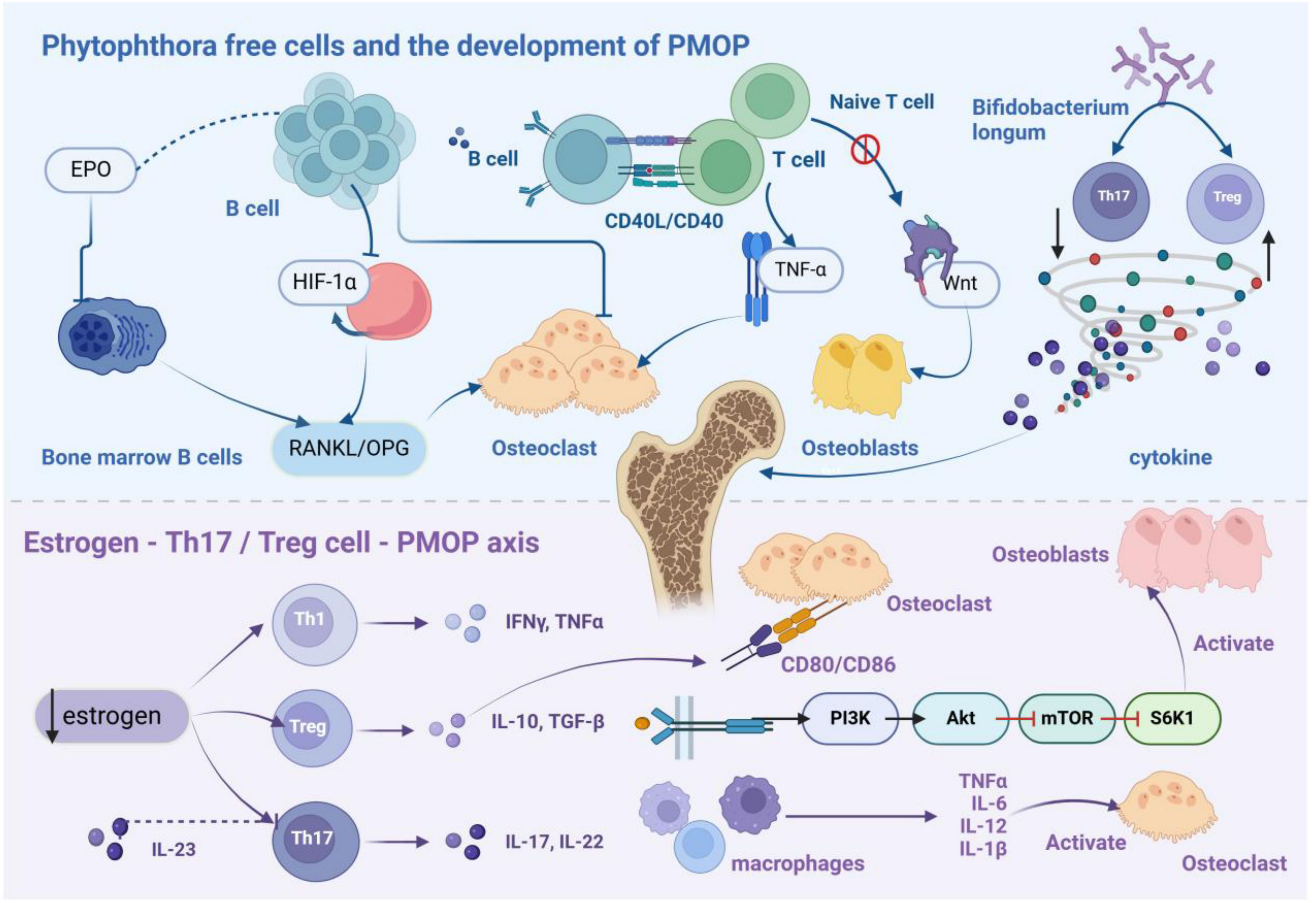
Immunopathological Mechanism of PMOP. B cells differentiate into osteoclasts, leading to bone loss. In the estrogen-deficient state, hypoxia-inducible factor-1α signaling is activated in B cells, enhancing RANKL expression and promoting osteoclastogenesis, thereby inducing PMOP. T cells normally exhibit bone-protective functions in basal bone metabolism. However, following ovariectomy, CD4+ and CD8+ T cells become activated and secrete RANKL and other osteoclastic factors. Treg cells secrete IL-10, TGF-β, and other anti-resorptive cytokines, whereas Th17 cells produce IL-17, which stimulates osteoclastogenesis. Additionally, IL-17 triggers mesenchymal stem cells to release osteoclast differentiation factors. Thus, estrogen modulates bone metabolism by regulating the balance between Th17 and Treg cells.

PMOP is primarily caused by estrogen deficiency, which leads to decreased bone mass, deterioration of microarchitecture, and impaired bone metabolism. A hallmark pathological feature of PMOP is the imbalance between bone resorption and formation, with accelerated resorption outpacing bone formation (14). Recent advances in osteoimmunology have revealed that estrogen deficiency induces a chronic low-grade inflammatory state, significantly contributing to disease progression. This inflammatory response is mediated through various immune pathways, driving the pathological mechanisms underlying PMOP. System is shown in **Figure 1**.

### 2.1 Immune cells and the development of PMOP

B cells contribute to humoral immunity through antibody production and regulate bone metabolism. Studies have highlighted their osteoclastogenic potential, particularly under erythropoietin stimulation, where bone marrow B cells express receptor activator of nuclear factor kappa-Β ligand (RANKL), modulating bone metabolism via the RANKL/osteoprotegerin axis. High erythropoietin concentrations induce B cell differentiation into osteoclasts, resulting in bone loss (15). Additionally, estrogen downregulates hypoxia-inducible factor-1α expression by upregulating heat shock protein 70 production. In ovariectomized mice, elevated hypoxia-inducible factor-1α levels activate downstream pathways, upregulating RANKL gene expression in B cells and promoting osteoclastogenesis, thus accelerating PMOP progression (16).

T cells, originating from bone marrow lymphoid stem cells, mature in the thymus before circulating through systemic immune organs and tissues. Under normal conditions, T cells maintain bone homeostasis via multiple mechanisms. Estrogen presence facilitates T cell-derived CD40 ligand interactions with B cell surface CD40, enhancing osteoprotegerin mRNA expression and protecting bone (17). However, in PMOP, this protective mechanism is disrupted. Following ovariectomy, activated CD4+ and CD8+ T cells increase Dickkopf-1 production, inhibiting osteoblast Wnt signaling via paracrine effects (18). Simultaneously, these T cells secrete tumor necrosis factor-α (TNF-α) and RANKL, accelerating bone resorption (19). PMOP patients also exhibit elevated LIGHT expression in circulating monocytes and T cells, which enhances osteoclastogenesis by modulating TNF and RANKL expression (20).

Treg cells and Th17 cells, key subsets of T cells, exhibit differentiation plasticity but serve opposing functions. Treg cells suppress osteoclast differentiation by inhibiting IL-17 expression, whereas Th17 cells promote osteoclastogenesis via RANKL signaling (21). Maintaining the balance between these populations is critical for preserving normal bone mass (22). Emerging research demonstrates that gut microbiota significantly influences Treg and Th17 cell functions. For example, *Bifidobacterium longum* modulates Breg cell expression, establishing a Breg-Treg-Th17 axis that enhances Treg cell function and suppresses Th17 activity. This modulation reduces pro-inflammatory cytokines, such asInterleukin-6 (IL-6), Interleukin-17 (IL-17), and TNF-α, while increasing anti-inflammatory factors, including Interleukin-10 (IL-10) and IFN-γ, thereby protecting bone and alleviating PMOP symptoms (23).

### 2.2 The estrogen-Th17/Treg cell-PMOP axis

Chronic inflammation-induced bone metabolic dysregulation is a fundamental pathological feature of PMOP, with the Th17/Treg cell balance and associated cytokine networks orchestrating bone remodeling. Treg cells inhibit bone resorption through two mechanisms: secretion of anti-resorptive factors such as IL-10 and transforming growth factor-β (TGF-β), and interaction of their surface cytotoxic T-lymphocyte-associated protein 4 with CD80/CD86 on osteoclast precursors, which activates indoleamine-2,3-dioxygenase. This activation triggers tryptophan catabolism, inducing precursor cell apoptosis (24). Conversely, Th17 cells enhance osteoclastogenesis by expressing RANKL and secreting IL-17, which stimulates macrophages to produce pro-inflammatory mediators, including TNF-α and IL-6, further upregulating RANKL expression in osteoclast-supporting cells (22).

Estrogen deficiency disrupts the Th17/Treg balance. In estrogen-depleted conditions, heightened Th17 cell activity increases IL-17 production, promoting bone marrow mesenchymal stem cell proliferation and osteogenic differentiation while inducing macrophage colony-stimulating factor and RANKL secretion, thereby accelerating osteoclastogenesis (22, 25). Clinical studies report significantly elevated IL-17 levels in postmenopausal osteoporotic vertebral compression fracture patients (26). Additionally, Interleukin-23 exacerbates bone loss through two mechanisms: enhancing Th17 cell activity and inducing T cell RANKL expression. Targeting these pathways offers therapeutic potential, as anti-IL-17 antibodies promote bone regeneration via forkhead box o1 and activating transcription factor 4 activation (27), while anti-Interleukin-23 antibodies prevent estrogen deficiency-induced bone loss (28). Recent studies show that B10 cell adoptive transfer reduces Th17 cell populations and inhibits alveolar bone osteoporosis in ovariectomized mice (29). At the molecular level, estrogen stimulates Treg cells to produce IL-10 and TGF-β1, suppressing osteoclast differentiation and bone resorption. TGF-β, a key bone repair regulator, balances osteoblast differentiation and osteoclast formation (30). Molecular studies reveal that TGF-β1 enhances osteoblast survival, differentiation, and migration by activating the phosphatidylinositol 3-kinase/protein kinase B/mechanistic target of rapamycin/ribosomal protein S6 kinase beta-1 signaling pathway (31). These findings suggest that targeting Treg cell activity may provide effective therapeutic strategies for bone protection in inflammatory conditions.

## 3 Intestinal homeostasis affects the development of PMOP

PMOP is the most common form of primary osteoporosis and is characterized by its complex pathogenesis. Recent research has underscored the critical role of the gut microbiota in the development and progression of this condition. PMOP is associated with significant alterations in gut microbiota composition, including an inverse relationship between the abundance of *proteobacteria* and bone mass. Additionally, the relative abundances of *bacteroides*, *parabacteroides*, and lactobacillus are significantly increased. These microbial changes are thought to aggravate bone loss by influencing bone metabolism and promoting inflammatory responses, thus accelerating disease progression.The gut microbiota is a critical regulator of immune system homeostasis, modulating immune cell activity and cytokine networks (32–35). Accumulating evidence indicates that gut microbiota dysbiosis significantly contributes to the pathogenesis of PMOP, primarily by disrupting the Th17/Treg cell balance. This immunological equilibrium is precisely governed by the gut microbiota and its metabolic products (36, 37).

The gut microbiota influences PMOP through two distinct immunological mechanisms. In adaptive immunity, specific bacterial populations exhibit unique immunomodulatory functions. For instance, *segmented filamentous bacteria* (*SFB*) promote intestinal Th17 cell development through defined pathways, with *SFB*-mediated Th17 responses enhancing barrier integrity by inhibiting bacterial translocation in constitutively myosin light chain kinase-activated mouse models (38). Notably, intestinal Th17 cells display significant functional heterogeneity: *SFB*-induced Th17 cells maintain gut homeostasis, while *citrobacter*-induced Th17 cells exhibit pro-inflammatory characteristics (39). Additionally, *clostridium* species produce immunomodulatory metabolites that stimulate Treg cell development, thereby establishing immune tolerance (40–42).

**Figure 2:**
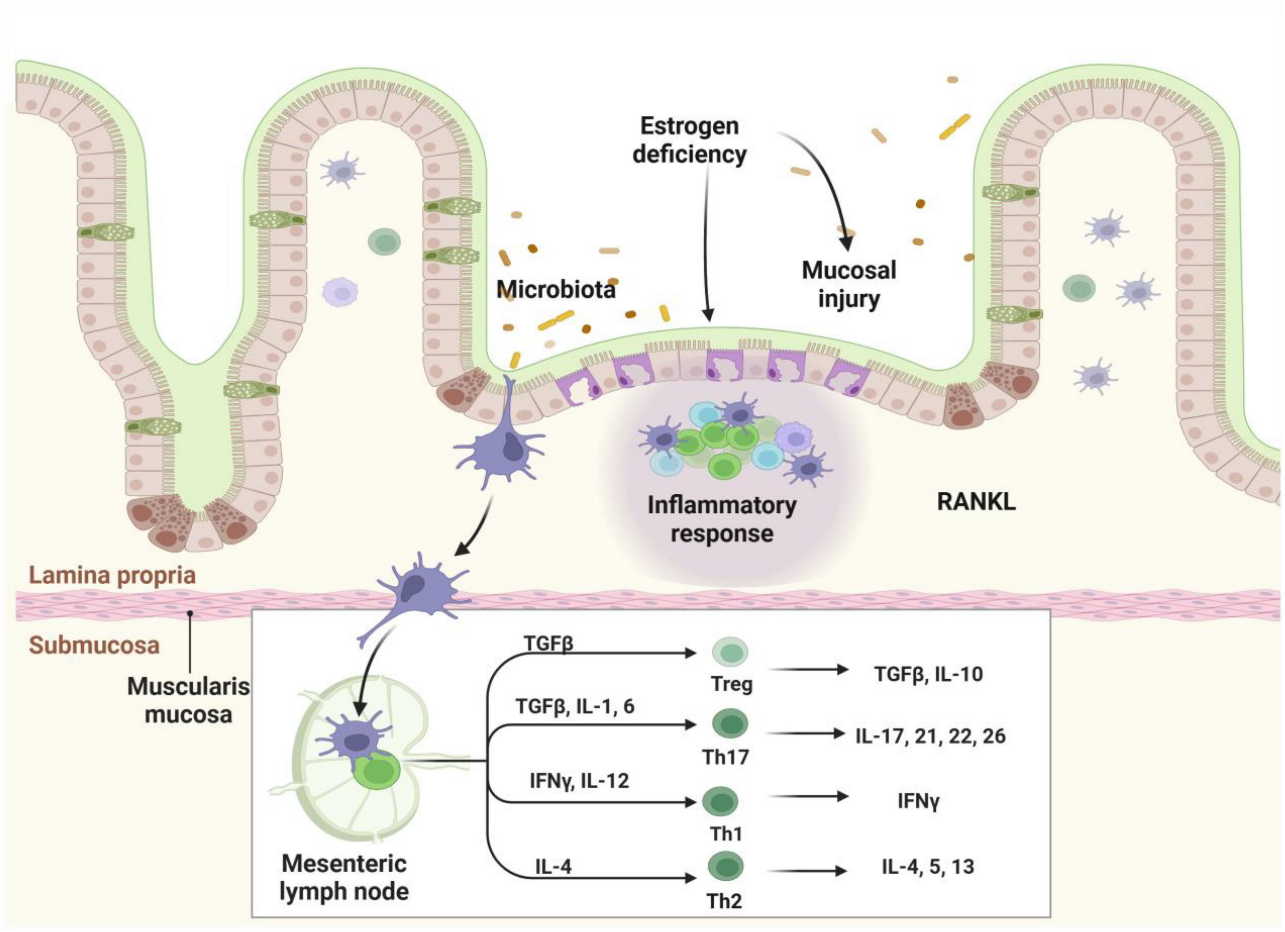
Gut Homeostasis and Immune Regulation. Gut homeostasis plays a pivotal role in maintaining immune equilibrium. Disruption of the gut microbiota and damage to the intestinal barrier can lead to immune dysregulation, characterized by the abnormal activation of innate immune cells, including dendritic cells and macrophages. This activation induces the production of elevated levels of pro-inflammatory cytokines, such as IL-12, IL-23, and type I interferons, while anti-inflammatory cytokines, including TGF-β and IL-10, are suppressed. Activated antigen-presenting cells, like dendritic cells and macrophages, present microbial antigens to CD4+ T helper cells, driving their differentiation into various pro-inflammatory T cell subsets, including Th1, Th2, and Th17.

In innate immunity, gut-associated lymphoid tissue-resident cells serve as the primary defense against exogenous antigens. Disruption of gut microecological homeostasis abnormally activates innate immune cells, increasing pro-inflammatory mediators such as IL-12, Interleukin-23, and type I interferons and reducing anti-inflammatory factors such as TGF-β and IL-10 (36). This dysregulation enhances antigen presentation to CD4+ T cells by dendritic cells and macrophages, promoting differentiation into inflammatory T cell subsets (43). Under specific microenvironmental conditions, CD4+ T cells can alternatively differentiate into immunosuppressive Treg cells, forming complex regulatory networks (44, 45). System is shown in **Figure 2**.

The gut microbiota also regulates bone metabolism through endocrine signaling pathways. Intestinal epithelial cells activate mitogen-activated protein kinase signaling via estrogen receptors (46), triggering nitric oxide/cyclic guanosine monophosphate cascades that enhance osteoblast differentiation and osteoprotegerin secretion while inhibiting osteoclastogenesis (47). Furthermore, *clostridium* species maintain estrogen homeostasis through β-glucuronidase-mediated enterohepatic circulation (48), directly linking estrogen levels to microbiota composition. During PMOP progression, the gut microbiota modulates disease development by influencing estrogen deficiency-induced inflammation. Decreased estrogen levels impair intestinal barrier integrity, enhancing Th17 cell differentiation. These activated Th17 cells produce pro-inflammatory mediators such as TNF-α, RANKL, and IL-17, creating a positive feedback loop that promotes osteoclastogenesis and bone loss (49). These mechanisms highlight the therapeutic potential of targeting the gut microbiota-immune-endocrine axis in PMOP treatment.

## 4 Mechanisms by which gut homeostasis regulates the Th17/Treg cell balance and its impact on PMOP

**Figure 3:**
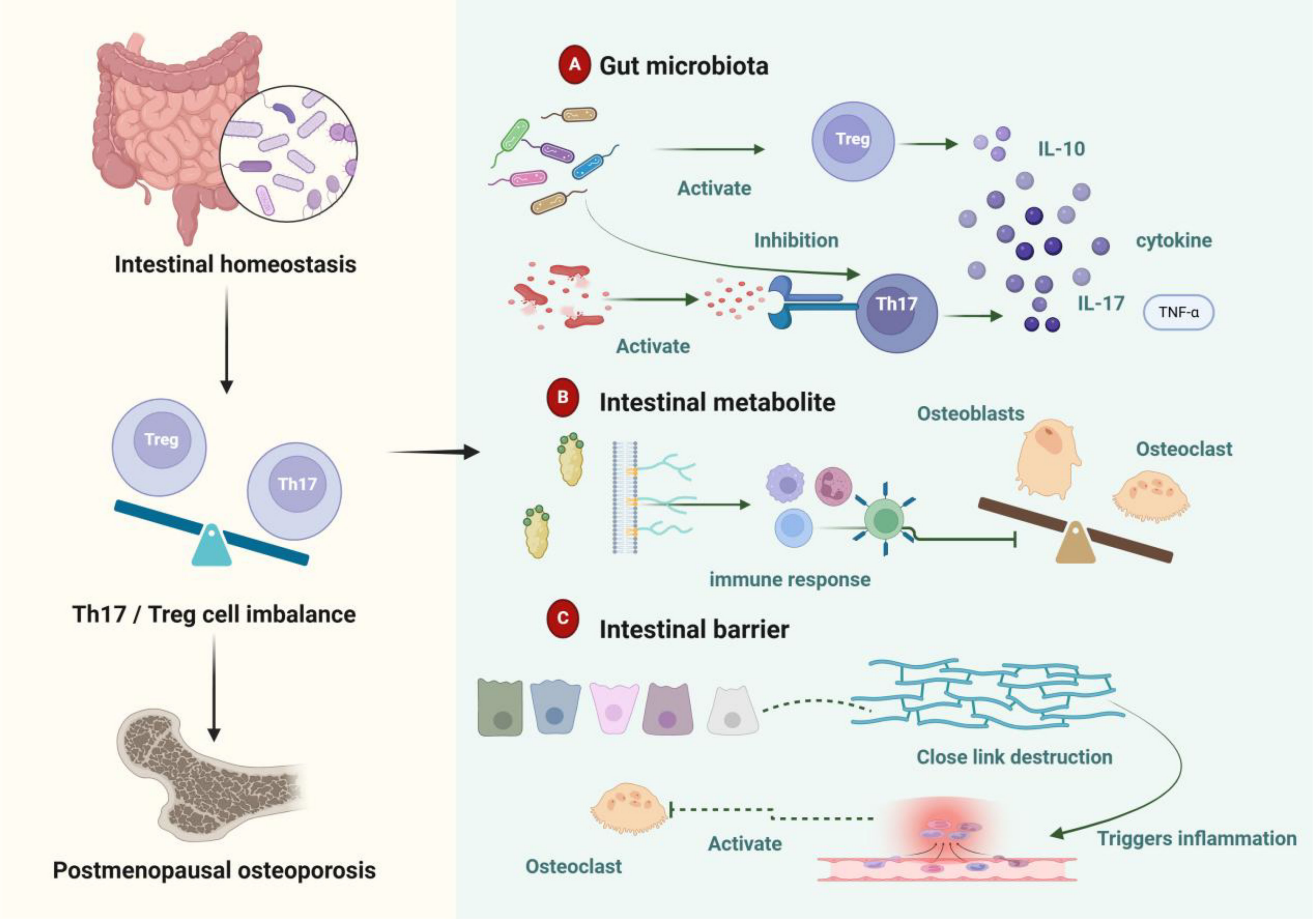
Gut Homeostasis Regulates Th17/Treg Balance and Influences PMOP. **(A)** The gut microbiota modulates the balance between Th17 and Treg cells, as well as cytokine levels, affecting the development and progression of PMOP. **(B)** Microbial-derived metabolites from the gut regulate multiple immune cells, causing an imbalance between osteoblasts and osteoclasts, which contributes to PMOP. **(C)** Intestinal barrier damage increases permeability and disrupts tight junctions, triggering inflammation and promoting osteoclast differentiation, thereby accelerating the onset and progression of PMOP.

Gut homeostasis is a multi-layered defense system comprising the gut microbiota, mucus layer, single-layer epithelium, and immune cells within the lamina propria (50). The mucus layer and epithelial cells form a physical barrier that prevents bacterial adhesion (51). The lamina propria and submucosa play critical roles in immune responses, protecting the host from both commensal and pathogenic microbial invasion (52). Epithelial cells regulate trans-epithelial permeability through tight junctions, thereby maintaining epithelial barrier integrity (53, 54). Disruption of gut homeostasis in PMOP is primarily mediated by the immune-modulating effects of microbial metabolites (55, 56). When gut homeostasis is compromised, pathogenic microbes proliferate, and excessive production of metabolites such as *lipopolysaccharides* (LPS) damages the intestinal barrier. This increases mucosal permeability and exacerbates both intestinal and systemic inflammatory responses. Under these conditions, Th17 cell activity is elevated while Treg cell function is suppressed, creating an imbalance that accelerates osteoclast formation and bone resorption. This process ultimately contributes to bone loss and PMOP progression. **Figure 3** illustrates the impact of gut homeostasis on the Th17/Treg balance in PMOP.

### 4.1 The gut microbiota-Th17/Treg-PMOP axis

In patients with PMOP, an increased abundance of *clostridium* species has been observed, with its relative abundance negatively correlated with bone mineral density (BMD) (57). Research suggests that *clostridium* activates the protein kinase B beta signaling pathway, promoting M1 macrophage production, thereby triggering inflammation and accelerating osteoporosis progression (58, 59). In a randomized trial involving postmenopausal Japanese women, participants treated with the probiotic *Bacillus subtilis* showed a significant increase in total hip BMD compared to the placebo group (60). After 12 and 24 weeks of *Bacillus subtilis* treatment, the abundance of *clostridium* species significantly decreased, potentially improving BMD by reducing pro-resorptive cytokines (60). Additionally, *lactobacillus reuteri* (american type culture collection PTA-6475) has been shown to reduce bone loss in elderly women with low BMD (61).

*Lactobacillus acidophilus* mitigates bone loss and enhances bone heterogeneity in osteoporotic mice by modulating the Treg-Th17 cell balance (62). Similarly, *lactobacillus rhamnosus* reduces bone loss and preserves bone health in ovariectomized mice (63). Probiotics such as *Bifidobacterium* and *clostridium* species promote Treg cell differentiation, thereby inhibiting excessive bone resorption. Intervention with *Bifidobacterium longum* significantly increases Breg cell proportions and the levels of IL-10 and interferon-gamma while decreasing the production of TNF-α, IL-6, and IL-17. Clostridium-stimulated Bregs also significantly enhance Treg cell proportions and IL-10 expression while reducing Th17 cells and IL-17 levels, indicating a potent regulatory role in Th17/Treg differentiation. Pathogenic bacteria such as *clostridium* and *Ruminococcus* are associated with Th17 cell activation, exacerbating osteoclastogenesis and bone resorption (64). Toxins produced by *Bacteroides fragilis* activate intestinal Th17 cell recruitment via the JAK-STAT3 pathway (65–67). Thus, alterations in gut microbiota composition disrupt the Th17/Treg balance, affecting bone metabolism and contributing to osteoporosis progression (68).

### 4.2 The gut metabolites-Th17/Treg-PMOP axis

Gut microbiota-derived metabolites play a pivotal role in regulating immune cell development and function, including both adaptive and innate immune responses. IL-10, produced by effector T cells, acts as a key self-regulatory mechanism for maintaining immune homeostasis (68). Short-chain fatty acids (SCFAs) enhance IL-10 production in differentiated Th1 cells via a G-protein-coupled receptor 43-dependent pathway and induce IL-10 during Th1 and Th17 cell differentiation by inhibiting histone deacetylase activity (69). SCFAs and other metabolites directly influence osteoblast and osteoclast differentiation and activation, with butyrate playing a crucial role in osteoclast metabolism regulation (33). Butyrate-treated dendritic cells induce the expression of immunosuppressive enzymes, such as indoleamine 2,3-dioxygenase 1 and aldehyde dehydrogenase 1 family member A2, in a solute carrier family 5 member 8-dependent manner. This promotes Treg differentiation while inhibiting Th1 differentiation (70). Microbial tryptophan metabolites, such as indole and its derivatives, bind to aryl hydrocarbon receptors, influencing B cell development (71), differentiation (72), and cytokine regulation (73) via aryl hydrocarbon receptor signaling. LPS, components of Gram-negative bacteria, trigger cytokine cascades driving T cell-mediated inflammation (74). LPS also inhibits osteoblast maturation and activates osteoclasts, enhancing bone resorption and exacerbating PMOP progression. Additionally, bile acids and their metabolites regulate host immune responses by modulating the Th17/Treg cell balance (75). These metabolites provide mechanistic insights into the interplay between gut homeostasis and immune regulation.

### 4.3 The gut barrier-Th17/Treg-PMOP axis

Disruption of gut barrier function can lead to leakage of intestinal contents, creating a pro-inflammatory environment (76). In mouse models, elevated zonulin levels degrade essential tight junction proteins and increase the expression of claudin-2 and claudin-15, compromising tight junction integrity and severely impairing gut barrier function Increased intestinal permeability triggers T cell-mediated mucosal inflammation and facilitates the migration of autoreactive T cells, such as Th1 and Th17 cells, from the gut to other sites like joints, potentially contributing to PMOP onset (77). Estrogen plays a key role in maintaining intestinal epithelial barrier function, and its deficiency increases gut permeability (78). This enhanced permeability triggers inflammatory responses that promote osteoclast formation, ultimately leading to bone loss (79, 80). Sufficient estrogen levels activate Tregs, inhibit osteoclastogenesis, and prevent osteoblast destruction, contributing to the maintenance of bone mass (79, 81, 82). Tight junctions between intestinal epithelial cells are essential for preserving gut barrier integrity. *Lactobacillus rhamnosus* supports epithelial integrity through carbohydrate transport and metabolism (83). Specifically, *lactobacillus rhamnosus GG* regulates both the gut microbiota and gut barrier, modulating the Th17/Treg balance and alleviating osteoporosis induced by estrogen deficiency.

## 5 PMOP treatment strategies based on gut homeostasis regulation

Current clinical treatments for PMOP, including calcium supplements, calcitonin, bisphosphonates, and estrogen, often have limited efficacy and are associated with significant side effects. Consequently, there is a pressing need for safe, effective, and low-risk therapeutic options for PMOP patients. In recent years, increasing research into the relationship between gut microbiota and bone metabolism has highlighted the potential of strategies targeting gut homeostasis for preventing and treating PMOP. The interaction between gut microbiota and bone metabolism offers a novel approach to osteoporosis management. By modulating the host’s immune, metabolic, and endocrine systems, the gut microbiota can directly or indirectly regulate bone homeostasis, presenting new possibilities for the comprehensive treatment of PMOP.

### 5.1 Gut microbiota modulators

Probiotics have demonstrated potential to enhance the growth and metabolic activity of beneficial bacteria, offering significant benefits for bone health (16). They produce metabolites and genetic products that directly interact with epithelial and immune cells, improving gut function, reducing inflammation, lowering intestinal pH to promote calcium absorption, and preventing the colonization of harmful bacteria. Collectively, these effects support osteoblast activity and contribute to the maintenance of bone health (84, 85). Probiotic supplementation has been shown to increase bone density and promote fracture healing. Animal studies have further demonstrated that probiotics enhance bone mass by inhibiting CD4+ T cell proliferation in the bone marrow and reducing the expression of pro-inflammatory cytokines such as TNF-α (11, 86).

Additionally, the gut microbiota regulates immune balance and microbial stability through tryptophan and its metabolites, including indole and serotonin (87). Exogenous supplementation of tryptophan metabolites, such as indole acetic acid and indole-3-propionic acid, effectively restores intestinal barrier integrity in ovariectomy -induced PMOP mouse models and alleviates osteoporosis. This process critically depends on the activation of the aryl hydrocarbon receptor. Mechanistically, tryptophan metabolites, particularly indole acetic acid, activate intestinal AhR, which, in turn, stimulates the Wnt/β-catenin signaling pathway to restore intestinal barrier function. indole acetic acid and indole-3-propionic acid supplementation also enhances M2 macrophage secretion of IL-10, which diffuses from the intestinal lamina propria to the bone marrow, promoting osteogenesis while suppressing osteoclast formation. Notably, the therapeutic effects of tryptophan metabolites on intestinal homeostasis and osteoporosis symptoms are significantly reduced in ovariectomy mice lacking intestinal AhR. These findings highlight gut microbial tryptophan metabolites as promising therapeutic candidates for osteoporosis by modulating the AhR-mediated gut-bone axis.

By modulating the immune system, the gut microbiota influences bone metabolism, potentially affecting bone density and turnover. This highlights the gut microbiota as a novel therapeutic target for osteoporosis treatment and fracture prevention (5). For example, *Lactobacillus casei* modulates gut microbiota composition, reduces pro-inflammatory cytokines such as IL-17, interleukin-1β, IL-6, and TNF-α, and adjusts the Th1/Th17 ratio, thereby inhibiting the onset and progression of PMOP (88). Another byproduct of colonic microbial fermentation, hydrogen gas, is produced in significant amounts by certain strains of *clostridium* and may have potential effects on gut and bone health (89–91).

### 5.2 Fecal microbiota transplantation

Fecal microbiota transplantation (FMT) has been shown to reshape gut microbiota and mitigate bone loss in ovariectomy-induced osteoporotic mice. The mechanisms underlying these effects include correcting gut microbiota dysbiosis, elevating SCFAs levels, improving gut permeability, and inhibiting the release of pro-osteoclastogenic factors, collectively suppressing excessive osteoclast formation (92). The circulatory system serves as a bridge connecting osteoclastogenic factors, transgenic cells, SCFAs, and the skeletal system. FMT effectively prevents ovariectomy-induced bone loss by limiting osteoclast overactivity (93). Compared to ovariectomized controls, FMT-treated mice exhibited increased expression of tight junction proteins such as occludin and reduced secretion of pro-osteoclastogenic factors, including TNF-α and interleukin-1β (94). Furthermore, FMT optimized gut microbiota composition and abundance, while increasing fecal SCFAs levels, particularly acetate and propionate (95). Consequently, FMT represents a promising alternative therapy and a potential strategy for preventing and treating PMOP in the future.

### 5.3 Maintaining gut barrier integrity

The integrity of the gut barrier is essential for the prevention and treatment of PMOP. An intact mucosal barrier prevents harmful substances, such as bacterial LPS, from entering systemic circulation (94). This, in turn, suppresses activation of the toll-like receptor 4 receptor signaling pathway in macrophages, thereby reducing the release of pro-inflammatory cytokines like TNF-α and inhibiting osteoclast differentiation and activation, which would otherwise accelerate bone resorption (96, 97). Additionally, a healthy gut microbiota produces beneficial metabolites, including SCFAs, that regulate bone metabolism. SCFAs inhibit osteoclast activity by downregulating key molecules such as TNF receptor-associated factor 6 and nuclear factor of activated T-cells 1, while simultaneously promoting bone formation by upregulating osteoblast differentiation-related genes (61, 98). Butyrate, a major SCFAs, promotes the differentiation of bone-protective Tregs, which, in turn, induce CD8+ T cells to release Wnt10b, activating the Wnt signaling pathway in osteoblasts (99, 100). Lucas et al. demonstrated that SCFAs supplementation or a high-fiber diet can prevent menopause and inflammation-induced bone loss, significantly increasing bone mass (70). Vegetarians and individuals adhering to a mediterranean diet tend to have higher SCFA levels, which are associated with improved bone health (101). Dietary supplementation with oligosaccharides enhances SCFA production, contributing to increased BMD (102). In antibiotic-treated mice, SCFA supplementation reduced bone loss without affecting bone turnover rates (103). Thus, maintaining gut barrier integrity and regulating gut microbial metabolites are critical for inhibiting bone resorption and promoting bone formation effectively.

## 6 Conclusion

PMOP is a complex metabolic bone disorder characterized by estrogen deficiency, which leads to bone loss and structural deterioration through disruptions in the immune-endocrine network. Recent research highlights the central role of intestinal homeostasis in PMOP development by regulating immune and endocrine systems. Dysregulation of the gut microbiota-Th17/Treg-bone metabolism axis has emerged as a key pathogenic mechanism. Disrupted microbial balance alters Th17/Treg homeostasis, stimulates osteoclast formation, and increases bone resorption, ultimately compromising bone integrity. Microbial metabolites influence bone cell function through immune activation and Th17/Treg modulation, driving disease progression. Impaired gut barrier function further initiates inflammation, accelerating bone loss.

These findings suggest that therapeutic strategies targeting gut microbiota and barrier function could regulate metabolic and immune systems, offering new opportunities for PMOP treatment. However, the specific mechanisms through which microbial species or metabolites regulate signaling pathways and immune responses in PMOP remain poorly understood, and their clinical potential requires further validation. Future research should prioritize clinical studies exploring the efficacy and mechanisms of gut microbiota regulation in PMOP prevention and treatment. Investigating the roles of specific probiotics or metabolites, developing novel gut microbiota-targeted therapies, and combining traditional pharmacological treatments with lifestyle interventions could provide innovative approaches for the comprehensive management of PMOP. As the field of osteomicrobiology advances, future studies should also examine the interactions between gut microbiota and drug metabolism in PMOP patients, as well as the potential of gut homeostasis regulation in PMOP therapy. These efforts could lead to novel therapeutic strategies and targets, better addressing patient needs, improving treatment outcomes, and enhancing quality of life.
